# From Quality Detection to Intelligent Management: A Mechanism-Informed Framework Integrating Foodomics, Microbiome Science and Explainable Artificial Intelligence for Fish Flesh Quality

**DOI:** 10.3390/foods15214007

**Published:** 2026-11-09

**Authors:** Jiangqi Qu, Ran Guo, Guang Chen, Jingjing Guo, Qiuzhen Wang

**Affiliations:** 1 Fisheries Science Institute, Beijing Academy of Agriculture and Forestry Sciences, Beijing 100068, China; 2 Ocean College, Hebei Agricultural University, Qinhuangdao 066000, China; qqzz1990@163.com; 3 Qinhuangdao Yier Biotechnology Co., Ltd., Qinhuangdao 066303, China; yierbio@126.com (G.C.); jingjing_445566@163.com (J.G.)

**Keywords:** fish flesh quality, foodomics, quality formation, gut microbiome, spoilage microbiota, machine learning, explainable artificial intelligence, harvest biological-state vector, quality prediction

## Abstract

Fish flesh quality is a multidimensional phenotype shaped across the production–post-harvest continuum by interacting metabolic, structural, lipid, redox, and microbial processes. Conventional sensory, physicochemical, biochemical, and instrumental measurements remain essential for defining quality phenotypes, while foodomics and microbiome approaches increasingly resolve the biological mechanisms underlying quality formation and deterioration. This review integrates recent advances in fish flesh quality assessment, foodomics, microbiome science, intelligent sensing, machine learning, and explainable artificial intelligence from a mechanism-informed perspective. Particular emphasis is placed on energy metabolism, lipid remodeling, redox regulation, muscle-fiber and collagen organization, microbial metabolites, and spoilage-related microbial functions that connect biological state with measurable quality traits. We further examine the transition from current-state recognition toward prospective quality prediction, with emphasis on biological interpretability, transferability, external validation, and uncertainty. Building on this evidence, we propose a dual-stage framework in which a context-dependent harvest biological-state vector integrates energetic, structural, lipid/nutritional, redox, and product-associated microbial dimensions to connect pre-harvest quality formation with post-harvest deterioration trajectories. Together, these advances provide a basis for moving from quality detection toward prospective prediction and mechanism-informed intelligent management of fish flesh quality.

## 1. Introduction

Aquatic foods have become increasingly important components of global food systems because they provide high-quality proteins, essential fatty acids, micronutrients, and other nutritionally valuable compounds while offering opportunities for more resource-efficient and sustainable food production [1,2]. Their growing recognition within the global “blue food” agenda further highlights their contribution to nutrition security, sustainable resource utilization, and resilient food systems [2,3]. At the same time, aquaculture has expanded rapidly as a major source of aquatic animal foods, driven by population growth, dietary transitions, and increasing pressure on capture fisheries [4,5]. As production continues to intensify, the priorities of aquaculture are extending beyond yield alone toward product quality, nutritional value, consumer acceptance, processing performance, and value retention throughout the production chain. Improving flesh quality has therefore become an increasingly important component of the transition from production-oriented aquaculture toward quality- and value-oriented development.

Fish flesh quality is inherently multidimensional and cannot be represented by a single indicator. It encompasses sensory properties, texture, water-holding capacity, color, nutritional composition, lipid quality, flavor-related characteristics, and muscle structural organization [6,7,8,9]. These traits emerge from interactions among tissue composition, physiological status, muscle development, nutrient allocation, environmental conditions, and post-harvest biochemical change [6,10]. Muscle-fiber characteristics and connective-tissue organization influence firmness, tenderness, and water retention, whereas intramuscular lipids and fatty-acid composition contribute to nutritional value, flavor potential, and oxidative stability [10,11]. Nutritional strategies, rearing conditions, and other production factors can further modify nutrient deposition, intermediary metabolism, muscle development, and flesh composition before harvest [10,12,13,14]. Fish flesh quality should therefore be viewed not simply as a set of measurable product traits but as the cumulative outcome of biological processes acting during growth, at harvest, and throughout post-harvest storage.

A broad range of sensory, physicochemical, biochemical, and instrumental approaches has been established to characterize these quality dimensions. Sensory and freshness assessment provides direct information on consumer-relevant attributes and deterioration status [15,16,17]. Texture analysis and water-holding-capacity measurements characterize physical and processing properties [6,7,8], whereas pH and nucleotide degradation indicate post-mortem biochemical progression [15,18,19]. Color assessment, compositional analysis, lipid indices, and oxidation markers provide additional endpoints for product comparison and quality control [12,20,21,22,23]. Advances in spectroscopy, imaging, electronic sensing, and other nondestructive technologies have further improved the speed, throughput, and objectivity of fish-quality assessment [24,25,26]. Importantly, these measurements are not purely descriptive. For example, pH decline and adenosine triphosphate (ATP) degradation can inform post-mortem energy metabolism, oxidation markers reflect redox damage, and water-holding capacity integrates changes in protein state, tissue structure, and water distribution. Their principal limitation is therefore not a lack of biological meaning but limited mechanistic specificity when individual endpoints are interpreted in isolation. Similar texture values may arise from different combinations of muscle-fiber organization, collagen properties, proteolysis, and oxidative injury, while comparable lipid contents may correspond to distinct fatty-acid profiles, metabolic states, and oxidative susceptibilities [6,9,11,14]. Conventional measurements thus remain indispensable as phenotype anchors and operational benchmarks, but additional biological resolution is needed to explain how these phenotypes arise and why they differ across species, production conditions, and post-harvest environments.

Foodomics provides one route toward this deeper biological resolution. By integrating metabolomics, lipidomics, transcriptomics, proteomics, and related molecular approaches, foodomics can connect measurable flesh quality traits with metabolic pathways, regulatory processes, and molecular intermediates [27,28,29]. Such approaches have increasingly been used to investigate energy metabolism, lipid remodeling, oxidative regulation, flavor-associated metabolites, muscle development, and other biological processes underlying quality variation [30,31]. Recent fish studies, interpreted within current food-metabolomics frameworks, have demonstrated the value of combining omics information with conventional physicochemical measurements to improve the interpretation of flesh quality, nutritional characteristics, and responses to production or post-harvest conditions [9,19,32,33,34]. The value of foodomics therefore lies not in replacing established quality measurements but in increasing the mechanistic resolution with which phenotypic differences can be interpreted. At the same time, high-dimensional molecular datasets create a translational challenge: biological signals identified in discovery studies must be validated, reduced to reproducible marker sets, and linked to measurable quality outcomes before they can support practical quality management.

Beyond host molecular regulation, microbial processes introduce an additional biological dimension because their roles differ fundamentally before and after harvest. Before harvest, the intestinal microbiome participates in nutrient transformation, digestive function, epithelial development, immune regulation, and host metabolism and may thereby influence nutrient utilization, physiological status, and muscle development [35,36,37]. Increasing evidence further links gut microbial and metabolic changes with edible-muscle phenotypes, including texture, collagen deposition, lipid metabolism, and nutritional composition [38,39,40,41,42]. After harvest, in contrast, product-associated microbiota act directly on edible tissue and contribute to quality deterioration through ecological succession, proteolysis, metabolite production, and the utilization of muscle proteins, lipids, nucleotides, and nitrogenous compounds [43,44]. These microbial compartments differ in anatomical location, available substrates, and functional significance and should therefore not be treated as interchangeable. Integrating microbiome information with host phenotypes and foodomics may help distinguish microbial processes associated with pre-harvest quality formation from those that directly drive post-harvest deterioration.

The increasing dimensionality of fish-quality data also creates a substantial analytical challenge. Machine learning and other artificial-intelligence approaches can identify nonlinear patterns and interactions within spectral, physicochemical, compositional, environmental, and multi-omics datasets [45,46]. In food-quality research, such models have improved the classification and prediction of freshness, composition, texture, and other product attributes [46,47], while applications in aquaculture and fisheries increasingly integrate biological, environmental, and production-related information for monitoring and decision support [48,49]. However, high predictive accuracy alone does not establish biological relevance, robustness, or transferability. Explainable artificial intelligence (XAI) can improve model transparency by identifying influential variables, spectral regions, or combinations of predictors [50,51,52], but these attributions require biological interpretation against independent physiological, biochemical, structural, or microbial evidence. Reliable intelligent quality management therefore depends not only on predictive performance but also on biologically appropriate validation, independence between training and test samples, external validation across production contexts, and careful interpretation of model outputs.

Taken together, current research has generated substantial knowledge on fish-quality phenotypes, molecular regulation, microbial processes, and intelligent prediction, yet these advances remain only partially integrated across the production–post-harvest continuum. A key unresolved issue is how biological processes shaping flesh quality before harvest are linked to the biochemical, structural, oxidative, and microbial trajectories that determine post-harvest quality fate. To address this gap, this review integrates conventional quality assessment, foodomics, microbiome science, and interpretable modeling to examine: **(i)** how biological and environmental processes shape flesh quality before harvest; **(ii)** how the biological state established at harvest influences subsequent deterioration; and **(iii)** how phenotypic, molecular, microbial, and sensing information can be translated into robust quality prediction and management. Building on this synthesis, we propose a dual-stage conceptual framework in which a context-dependent harvest biological-state vector links pre-harvest quality formation with post-harvest quality trajectories.

## 2. Literature Search, Review Scope and Evidence Terminology

### 2.1. Literature Search and Study Selection

The relevant literature was identified by searches of the Web of Science Core Collection, PubMed, Google Scholar, and SpringerLink. The main search period covered January 2000 through August 2026, and the search was last updated in August 2026. Earlier publications were retained when they provided foundational concepts or mechanistic evidence directly relevant to fish flesh quality, post-harvest deterioration, foodomics, microbiome research, or intelligent quality assessment.

The search was organized around four themes. Representative combinations included (“fish” OR “finfish”) AND (“flesh quality” OR texture OR “water-holding capacity”) AND (metabolomics OR lipidomics OR proteomics OR transcriptomics); (“fish” OR aquaculture) AND (“gut microbiome” OR microbiota OR “microbial metabolites”) AND (muscle OR “flesh quality”); (“fish” OR fillet) AND (freshness OR spoilage OR “microbial succession”) AND (storage OR preservation); and (“fish quality” OR freshness) AND (“machine learning” OR “deep learning” OR “hyperspectral imaging” OR spectroscopy OR “explainable artificial intelligence”). Search terms were adapted to the syntax of each database.

Priority was given to peer-reviewed studies that directly evaluated edible-muscle quality, mechanisms of pre-harvest quality formation, post-harvest biochemical or microbial deterioration, foodomics-based mechanism discovery, microbiome-associated regulation, or predictive modeling of fish-quality traits. Studies focused solely on pathogens or food safety without a quality or spoilage outcome, non-aquatic models without a foundational mechanistic role, and records lacking sufficient methodological detail were not used as core evidence. Evidence from crustaceans was included selectively when it provided complementary mechanistic insight relevant to processes also examined in finfish. Because this is a narrative review, the search strategy was designed for thematic coverage rather than formal systematic-review eligibility or meta-analysis.

### 2.2. Scope, Terminology and Evidence Interpretation

In this review, flesh quality refers to intrinsic and product-level characteristics of edible fish muscle, including texture, water-holding capacity, color, nutritional composition, flavor-related traits, and structural properties. Freshness describes the degree of post-mortem change during storage, whereas spoilage refers to biochemical and/or microbial deterioration that progresses toward sensory or technological unacceptability. Food safety is treated separately and concerns hazards such as pathogenic microorganisms, toxins, or contaminants. Stability refers to resistance to oxidative, structural, biochemical, or microbial deterioration, while processing performance includes technological properties such as yield, water retention, texture development, and gel-forming behavior.

Total viable count (TVC), also referred to as aerobic plate count in some studies, is interpreted as a culture-dependent measure of microorganisms capable of forming colonies under defined incubation conditions. It therefore does not represent total microbial abundance or microbial-community composition and cannot, by itself, identify specific spoilage organisms or establish food safety [15,53].

Because the literature considered in this review spans mechanistic intervention studies, comparative and multi-omics analyses, and broader cross-study synthesis, the strength of biological inference varies among reported relationships. For clarity, Level I evidence refers to relationships supported by controlled perturbation or intervention studies linking a biological process to a defined quality phenotype. Level II evidence refers to biologically coherent associations supported by comparative, longitudinal, or multi-omics observations without decisive causal manipulation. Level III evidence refers to integrative relationships proposed from cross-study synthesis and requiring further experimental validation.

## 3. Conventional Quality Assessment and Biological Interpretation of Fish Flesh Quality

Fish flesh quality is conventionally evaluated through sensory assessment, physicochemical measurements, and compositional analysis, which together provide essential phenotypic references for product comparison and quality evaluation [6,9,15]. Sensory and freshness approaches describe perceived acceptability and deterioration, whereas texture, water-holding capacity, and compositional assays quantify physical, processing, biochemical, and nutritional attributes [6,8,15]. These endpoints enable reproducible comparisons among dietary treatments, species, and production systems when analytical conditions are controlled [33,54]. Many of these measurements also carry biological information; however, individual endpoints often integrate several underlying processes and therefore provide limited specificity for identifying the dominant mechanism responsible for an observed phenotype [34].

The distinction between quality characterization and quality formation is therefore important for interpreting fish flesh quality data [9]. Similar texture values can result from different combinations of muscle-fiber development, collagen metabolism, oxidative injury, and post-mortem proteolysis [20]. Comparable total-lipid contents can likewise represent different nutritional value and storage stability because fatty-acid deposition, nutrient signaling, and lipid oxidation are biologically distinct processes [12,20]. Conventional indicators should therefore remain the primary phenotypic and operational benchmarks for quality assessment, while mechanistic interpretation is strengthened by integrating them with structural, biochemical, temporal, and molecular evidence.

### 3.1. Sensory Evaluation and Freshness Assessment as Operational Quality References

Sensory evaluation remains one of the most direct approaches for characterizing consumer-relevant fish-quality attributes, including appearance, odor, flavor, firmness, and overall acceptability [15,16,55]. Among sensory approaches, the Quality Index Method (QIM) converts characteristic deterioration attributes into demerit scores that increase during storage. In refrigerated mackerel, QIM changes were correlated with hardness, gumminess, chewiness and volatile basic nitrogen [17]. Similarly, application of species-specific QIM schemes to eight tropical fish species stored on ice yielded estimated shelf lives of approximately 8–18 days and revealed marked interspecific differences in ATP degradation and sensory rejection patterns [16]. These studies illustrate how sensory schemes provide operational reference points for tracking post-harvest freshness and defining product acceptability.

Freshness assessment is particularly informative because sensory deterioration integrates several post-mortem processes. Changes in appearance, odor, texture, and acceptability may reflect autolysis, nucleotide degradation, oxidation, microbial growth, and the accumulation of spoilage-related metabolites [15,18,25]. Sensory outcomes therefore represent biologically meaningful manifestations of deterioration rather than purely subjective descriptions. Their interpretation, however, is primarily related to the quality state of the product during storage. Traits established before harvest, including muscle-fiber architecture, collagen abundance, fatty-acid deposition, and metabolic composition, can influence subsequent sensory quality but are not individually resolved by a freshness score [20,54]. Consequently, fish with comparable sensory freshness may still differ in texture, nutritional composition, or flavor-precursor profiles because genotype, feeding strategy, rearing conditions, and exercise history shape muscle characteristics before harvest [20,56].

Species and product characteristics further influence the relationship between sensory deterioration and biochemical change [15,16]. In the eight-species comparison, K-values at sensory rejection ranged from approximately 30% to 80%, indicating substantial interspecific variation in nucleotide-degradation trajectories even under comparable iced-storage conditions [16]. QIM scores and related rejection thresholds are therefore most informative when calibrated for the relevant species, product form, and storage environment [15,17]. Sensory evaluation should consequently be regarded as an essential consumer-oriented and shelf-life endpoint that complements biochemical, structural, and instrumental measurements.

### 3.2. Instrumental Indicators and Their Mechanistic Interpretation

Instrumental measurements provide quantitative characterization of the physical, biochemical, nutritional, and appearance-related dimensions of fish flesh quality. Texture analysis and water-holding capacity (WHC) are among the most widely used physical measurements because they are directly related to eating quality, processing performance, and product yield. Hardness, springiness, cohesiveness, chewiness, and shear force describe different aspects of firmness and tenderness [7,54], whereas drip loss, cooking loss, and thawing loss characterize the ability of muscle to retain water during storage and processing [8]. These traits are biologically informative because they reflect the combined effects of muscle-fiber development, collagen organization, extracellular-matrix properties, myofibrillar integrity, proteolysis, and post-mortem structural change [43,54,56,57]. Their interpretation is therefore strengthened when physical measurements are considered together with histological, biochemical, or temporal information.

Color, pH and appearance-related measurements provide additional information on visual quality, market acceptance and post-harvest biochemical status. Color parameters such as L*, a* and b* are useful for describing appearance changes, but color variation may originate from multiple processes, including pigment composition, oxidation reactions, enzymatic or non-enzymatic browning, tissue characteristics and storage-related changes [21]. Muscle pH is closely linked to post-mortem glycolysis and acidification, whereas ATP degradation and the accumulation of adenosine diphosphate (ADP), adenosine monophosphate (AMP), inosine monophosphate (IMP), inosine, and hypoxanthine reflect nucleotide turnover and rigor-related biochemical progression [15,18,19]. These measurements can therefore provide relatively direct information on specific post-mortem processes, although their temporal patterns differ among species and are strongly affected by temperature, handling, and storage conditions [15,18]. Time-resolved measurements are particularly valuable because they distinguish dynamic deterioration trajectories from differences observed at a single sampling point.

Nutritional composition represents another major dimension of flesh quality assessment. Crude protein, total lipid, amino-acid profiles, and fatty-acid composition characterize nutrient deposition and nutritional value [12,20]. More detailed lipid indicators, including eicosapentaenoic acid (EPA), docosahexaenoic acid (DHA), ratios of polyunsaturated fatty acids (PUFAs) to saturated fatty acids (SFAs), lipid classes, and lipid-oxidation products, provide additional information on nutritional quality, flavor potential, and oxidative stability [12,20,22]. These compositional measurements are closely connected to biological processes such as nutrient allocation, lipid deposition, fatty-acid remodeling, and redox regulation [9,23]. Compositional and oxidation indicators are therefore particularly informative when interpreted together. Enrichment of unsaturated fatty acids, for example, may improve nutritional value while simultaneously altering susceptibility to lipid oxidation during storage [22,23]. Nutritional composition and oxidative stability should thus be considered related but non-equivalent components of flesh quality.

Nondestructive technologies have further expanded the capacity for rapid and high-throughput quality evaluation. Computer vision, Raman and near-infrared spectroscopy, electronic sensing, electrical impedance spectroscopy, and hyperspectral imaging can capture optical, spectral, spatial, volatile, or electrical features associated with freshness, composition, texture, and processing properties [15,58,59,60]. Their advantages include minimal sample destruction, rapid analysis, and potential integration into online grading or processing systems. The signals generated by these technologies commonly represent multivariate proxies of underlying chemical and structural properties rather than direct measurements of a single biological process. Their analytical value therefore depends on the choice of reference measurements, calibration strategy, validation population, and intended prediction target [15,58].

Taken together, instrumental measurements capture complementary dimensions of fish flesh quality rather than interchangeable aspects of a single quality axis. Texture, WHC, color, nutritional composition, oxidative stability, biochemical freshness, and processing performance may respond differently to the same biological or production intervention [7,8,12,20,22], while similar measured phenotypes may arise from different combinations of structural, metabolic, oxidative, and post-mortem processes [43,56,57]. Integrating these measurements therefore provides a more complete representation of the quality phenotype and increases the biological resolution available for interpretation. These phenotypic and biochemical reference points also provide the foundation for subsequent integration with foodomics, microbiome data, and predictive modeling. Table 1 summarizes the major approaches, the quality dimensions they capture, their practical applications, and the biological information they provide.

## 4. Foodomics-Driven Understanding of Fish Flesh Quality Formation and Improvement

Conventional quality measurements define the observable phenotype of fish flesh, whereas foodomics extends this information by linking measurable traits with the molecular processes through which nutritional, environmental, and post-harvest factors exert their effects [28,31]. By integrating transcriptomic, proteomic, metabolomic, and lipidomic information with structural, biochemical, and phenotypic measurements, foodomics can resolve molecular intermediates connecting controllable drivers with final product traits. Mechanistic interpretation is strongest when molecular responses are evaluated together with temporal changes, tissue structure, and defined quality outcomes.

**Figure 1:**
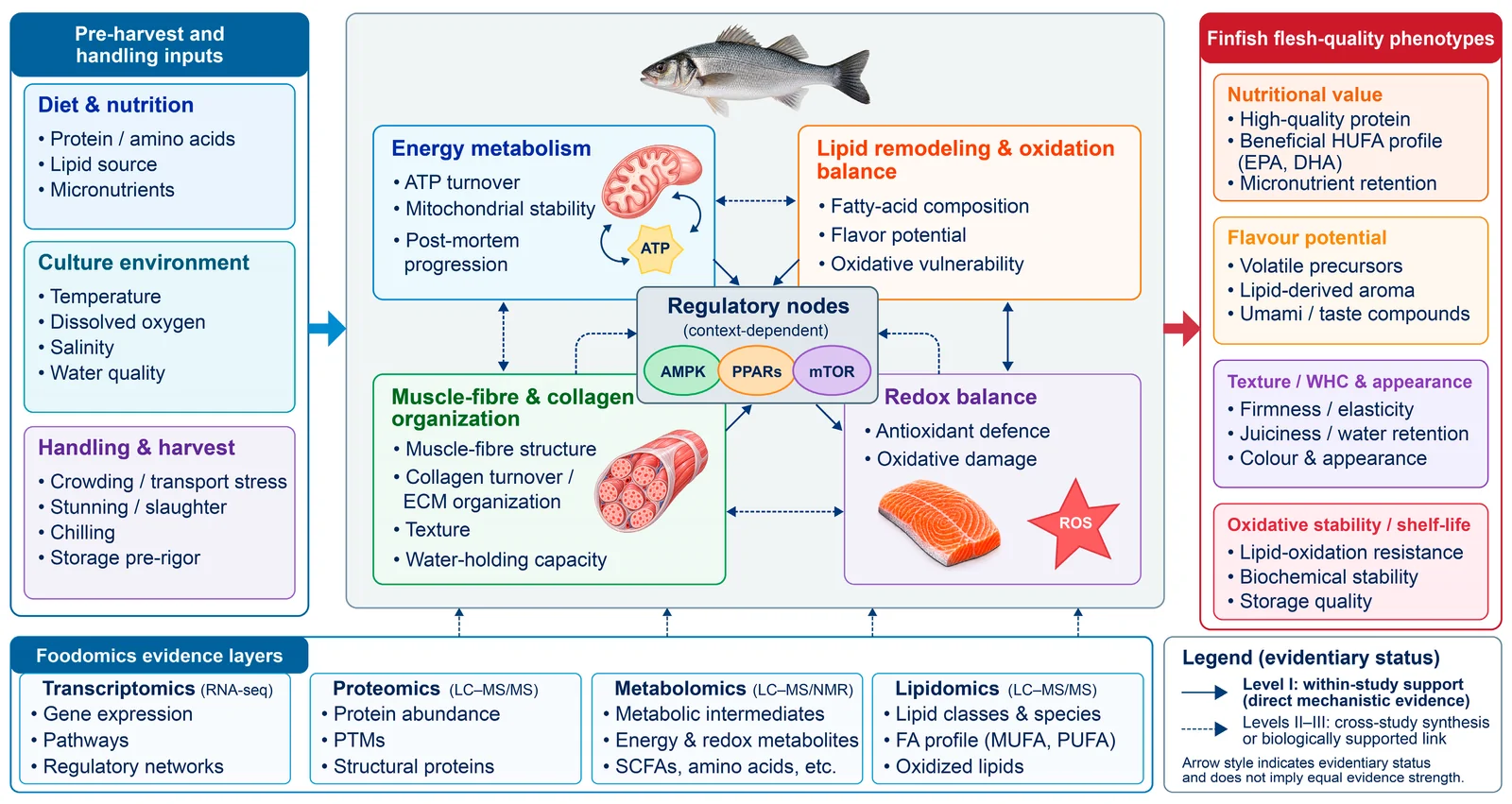
Foodomics-driven biological mechanisms associated with fish flesh quality formation and improvement. Foodomics integrates transcriptomic, proteomic, metabolomic, and lipidomic evidence with conventional flesh quality measurements. Solid arrows denote direct biochemical flows or relationships supported within intervention or mechanism-oriented studies, primarily corresponding to Level I evidence. Dashed arrows denote biologically supported associations, cross-study synthesis, or proposed links corresponding mainly to Levels II–III. Arrow style therefore indicates evidentiary status rather than implying that all displayed relationships are equally established.

Fish flesh quality emerges from coordinated metabolic, lipid, redox, and structural processes. Energy metabolism influences ATP turnover, mitochondrial stability, and post-mortem progression [18]. Lipid remodeling links fatty-acid enrichment with flavor potential and oxidative vulnerability [23], whereas muscle-fiber organization, collagen turnover, extracellular-matrix integrity, and post-mortem protein degradation contribute directly to texture and water retention [43,54]. Across these processes, AMP-activated protein kinase (AMPK), peroxisome proliferator-activated receptors (PPARs), and mechanistic target of rapamycin (mTOR)-related signaling repeatedly emerge as context-dependent regulatory nodes linking energy sensing, lipid metabolism, and structural growth [12,61,62]. Figure 1 integrates these relationships into a mechanistic framework connecting biological regulation with measurable flesh quality traits.

### 4.1. Energy Metabolism Links Physiological State with Post-Mortem Quality Stability

Energy metabolism provides an immediate biological connection between living muscle physiology and post-harvest flesh quality. After death, oxygen supply ceases, oxidative ATP production becomes restricted, anaerobic glycolysis consumes glycogen, lactate accumulates and pH declines. ATP depletion promotes actomyosin association, protein conformational change and proteolytic susceptibility, thereby affecting rigor development, water distribution, texture and shelf-life behavior [18]. Foodomics can further resolve whether deterioration is associated predominantly with altered nucleotide turnover, mitochondrial dysfunction, redox imbalance, or combinations of these processes before visible quality loss becomes apparent.

Post-mortem deterioration is increasingly understood as active biochemical progression rather than a passive decline in freshness. In black rockfish, rapid ATP depletion after death was accompanied by reactive oxygen species accumulation, calcium imbalance, cytochrome-c release, caspase-3 activation, lipid oxidation, myofibrillar degradation, discoloration, and texture loss [63]. Similar mitochondrial-apoptosis-related deterioration has been reported in other fish species. In northern pike, oxidative stress activated mitochondrial apoptotic factors and promoted cytoskeletal protein degradation during post-mortem storage [64]. In large yellow croaker, mitochondrial lipid alterations were associated with cytochrome-c release, caspase-3 activation, muscle oxidation and softening [65]. More directly, inhibition of mitochondrial-pathway apoptosis by cyclosporin A mitigated ATP decline, mitochondrial damage, cytochrome-c release, endogenous protease activation, myofibrillar protein degradation and lipid oxidation during refrigerated storage of large yellow croaker [66]. Together, these studies support mitochondrial integrity, redox balance and mitochondria-dependent apoptosis as mechanistic control points for post-mortem quality deterioration, although their practical value for preservation still needs validation using food-compatible interventions.

The metabolic state of muscle at harvest can itself be modified before death. Short-term micro-flowing-water treatment of crucian carp remodeled the tricarboxylic-acid cycle, purine metabolism, amino-acid metabolism, and fatty-acid metabolism together with improvements in flesh quality traits [19]. The involvement of purine metabolism is especially relevant because post-mortem ATP degradation proceeds through ADP, AMP, and IMP toward inosine and hypoxanthine [15,19]. Thus, foodomics can clarify whether a production intervention improves quality by reshaping metabolic readiness, enhancing flavor-related metabolites, or slowing post-mortem biochemical deterioration.

Energy metabolism also contributes to quality formation during muscle growth. In hybrid striped bass, better growth was associated with a greater proportion of small-diameter muscle fibers and stronger representation of glycolysis, the tricarboxylic-acid cycle, and amino-acid metabolism, whereas poor-growth fish showed stronger stress- and inflammation-related signatures [67]. Dietary inosine monophosphate in gibel carp activated hepatic AMPK signaling, improved hepatic lipid handling, and increased muscle IMP and lipid deposition together with improved flesh quality and flavor-related traits [62]. These findings indicate that edible quality can be shaped before harvest through interactions among energy supply, physiological stress, tissue growth, and nutrient partitioning.

Collectively, energy metabolism forms a mechanistic bridge between pre-harvest physiological state and post-mortem quality progression. ATP depletion, pH decline, nucleotide turnover, mitochondrial dysfunction, and oxidative stress represent interconnected components of this trajectory [18,19,63]. Pre-harvest factors such as flow regulation, exercise, and dietary intervention can modify the metabolic state of muscle before harvest [19,56], whereas handling and storage determine how this state subsequently evolves. Integrating energy-related pathways and metabolites with freshness, texture, water retention, and flavor-related nucleotide pools therefore provides a biological basis for understanding why products with similar initial phenotypes may follow different post-mortem trajectories.

### 4.2. Lipid Remodeling Connects Nutritional Enrichment with Oxidation Control and Sensory Quality

Lipid-related quality cannot be inferred from total lipid content alone. Nutritional value depends on lipid class and fatty-acid composition, particularly the deposition of long-chain n-3 polyunsaturated fatty acids [68]. However, the same unsaturated lipids that improve nutritional value may also increase oxidative susceptibility, and lipid oxidation products can interact with proteins, membranes and pigments during processing and storage [23]. Foodomics is therefore useful for distinguishing lipid accumulation from lipid remodeling and for separating beneficial nutritional enrichment from oxidative destabilization.

Dietary lipid source illustrates how nutritional deposition, structural quality and physiological context interact. In grass carp, fish oil increased muscle n-3 polyunsaturated fatty acids, including EPA and DHA, compared with soybean oil, and muscle fatty-acid composition closely reflected dietary input [69]. In largemouth bass, different lipid sources produced distinct patterns of n-3 PUFA deposition, collagen-related responses and texture traits; fish oil and rapeseed oil improved hardness, firmness, chewiness and springiness relative to palm oil [20]. The quality outcome also depends on dose and management context. Partial replacement of conventional oils with black-soldier-fly oil improved water-holding capacity and texture in largemouth bass, but the optimal response depended on substitution ratio [70]. In gibel carp, exercise altered lipid partitioning under a high-fat diet, reducing muscle fat, increasing fiber density and diameter, and improving inosine monophosphate, EPA and selected aroma volatiles when combined with dietary lipid intervention [43,56,57]. These studies show that lipid enrichment cannot be equated with overall quality improvement, because nutritional value, texture, flavor potential and storage stability may respond differently.

Lipid quality is also shaped by nutrient sensing and metabolic regulation beyond dietary fat itself. Dietary isoleucine in hybrid catfish changed muscle fatty-acid and amino-acid profiles through AMPK-associated SREBP-1c and PPARα signaling and autophagy-related protein turnover [12]. In Chinese mitten crab, an oleic-acid-rich finishing diet enhanced growth and antioxidant capacity and remodeled muscle lipid and amino-acid metabolism, with potential benefits for nutrient and flavor quality [71]. These findings support a broader view of lipid quality as a regulated metabolic state involving fatty-acid deposition, antioxidant capacity, amino-acid metabolism and protein turnover. For processing and storage, the key challenge is to enhance nutritional value without increasing oxidative vulnerability. Foodomics can support this balance by jointly assessing fatty-acid deposition, oxidized lipids, antioxidant metabolites, protein oxidation and flavor-related metabolites [23].

Protein-replacement studies further show how molecular profiling can help define formulation boundaries. *Clostridium autoethanogenum* protein could replace a substantial fraction of fishmeal without impairing growth or flesh quality, whereas complete replacement downregulated lipid metabolites and damaged intestinal structure [72]. High cottonseed-protein replacement reduced heat-insoluble collagen and hardness, increased drip loss and altered lipid and amino-acid metabolic pathways in largemouth bass [73]. These findings suggest that ingredient replacement may remain beneficial within a physiological tolerance range but could become detrimental once lipid metabolism, collagen integrity or intestinal function is disrupted. Foodomics can identify the formulation range at which metabolic and structural abnormalities emerge and nominate candidate processes underlying flesh quality loss [72,73], although time-resolved experiments are still required to determine which process fails first.

### 4.3. Muscle Fibers, Collagen and Extracellular Matrix Explain Structural Texture and Processing Performance

Texture and water-holding capacity are structural expressions of muscle organization, arising from the combined effects of myofibrillar proteins, sarcomere integrity, fiber size and density, connective-tissue collagen and extracellular-matrix organization. Because these components contribute differently to hardness, springiness, cohesiveness, shear force and water distribution, a single texture parameter cannot identify the dominant structural process responsible for a given phenotype [43,54]. Foodomics becomes informative when molecular changes are interpreted together with histology, collagen chemistry and instrumental texture, allowing mechanical traits to be linked with their biological origins.

Nutritional regulation can shape structural quality by altering fiber recruitment, collagen turnover and extracellular-matrix organization rather than merely changing measured firmness. In sub-adult grass carp, appropriate dietary protein increased hardness, promoted new fiber formation and enhanced collagen content, with collagen synthesis associated with TGF-β1/Smad and IGF-1/PI3K/AKT/p70S6K/LARP6 signaling and collagen degradation related to the TIMP-2/MMP-2 balance [54]. Collagen-targeted regulation is further supported by hydroxyproline supplementation studies. In Pacific white shrimp, dietary hydroxyproline increased collagen content and improved myofiber development, texture and taste evaluation [57]. In triploid crucian carp, hydroxyproline increased muscle collagen, collagen-synthesis gene expression and textural characteristics; the growth-derived optimum was approximately 0.74%, whereas several textural traits continued to increase at higher supplementation levels under the tested conditions [74]. These findings indicate that connective-tissue metabolism is a modifiable component of flesh texture, although effective supplementation depends on species, dose and the quality endpoint considered.

Protein source and feeding strategy further demonstrate that similar textural outcomes may arise through different structural and metabolic routes. In grass carp, dietary soybean-meal protein altered flesh quality together with myogenic regulatory factors, myosin-heavy-chain transcript profiles, fatty-acid metabolism, and oxidative phosphorylation, indicating coordinated effects on myogenic signaling, fiber characteristics, and metabolic phenotype [75]. In largemouth bass, both commercial and self-formulated feeds increased muscle hardness, shear force and fiber density and reduced fiber diameter compared with trash-fish feeding; however, increased heat-insoluble collagen and reduced drip loss were specifically reported for the self-formulated-feed group [34]. These findings demonstrate that similar improvements in instrumental texture do not necessarily arise from identical changes in collagen organization, fiber structure, or biochemical composition.

Structural quality can also deteriorate through different pre- and post-harvest routes. Dietary aflatoxin B1 in grass carp induced oxidative damage, reduced free amino acids and collagen, inhibited myogenic-regulatory genes and promoted extracellular-matrix degradation [14,43,54]. After harvest, spoilage bacteria degraded myofibrillar and connective-tissue proteins, damaged sarcomeres and worsened texture and water distribution [43,54]. These routes converge on structural quality loss but imply different points of intervention, including nutritional optimization, feed-hazard control, preservation, and microbial management. Integrating foodomics with texture and structural analysis can therefore help distinguish whether an unfavorable texture phenotype arises primarily from altered fiber development, collagen turnover, extracellular-matrix degradation, oxidative injury, or post-mortem proteolysis [14,43,54].

### 4.4. AMPK-, PPAR- and mTOR-Related Signaling as Recurrent Regulatory Nodes in Quality Formation

AMPK-, PPAR-, and mTOR-related signaling repeatedly emerge across nutritional and physiological studies as regulatory nodes connecting energy status, lipid metabolism, and structural growth. AMPK is a central cellular energy sensor that promotes ATP-generating pathways and contributes to mitochondrial and metabolic homeostasis [76,77]. In aquatic species, dietary IMP activated hepatic AMPK while improving liver lipid handling and increasing muscle IMP and lipid deposition in gibel carp [62]. Dietary isoleucine altered muscle fatty-acid profiles through AMPK-associated SREBP-1c and PPARα signaling in hybrid catfish [12]. These findings position AMPK-associated regulation at the interface between nutrient sensing, energy allocation, lipid metabolism, and the biochemical composition of edible muscle.

PPAR-related signaling provides a complementary lipid-remodeling branch. Foundational systems-biology evidence supports PPARα as a regulator of fatty-acid uptake, oxidation and lipid metabolic adaptation [78]. In grass carp, fish oil and soybean oil produced different muscle fatty-acid profiles and lipid-metabolic signaling [69]. Under oxidative stress, common-carp muscle showed altered PPAR signaling and lipid metabolism before bulk fatty-acid composition changed [79]. These findings support the use of PPAR-associated responses as indicators of lipid metabolic state, while also cautioning against treating PPARγ as a universal master regulator of aquatic flesh quality.

mTOR-related signaling links nutrient supply with protein deposition and structural growth. The general mechanistic literature describes mTOR as an integrator of amino-acid, insulin, IGF and growth signals that regulates protein synthesis and cell growth [80]. In hybrid bagrid catfish, appropriate isoleucine increased muscle mass and shifted fiber-diameter distribution while activating AKT/TOR/S6K1 and suppressing AKT/FOXO3a-associated protein degradation [61]. In grass carp, dietary protein altered hardness, fiber growth and collagen metabolism together with changes in IGF-1/PI3K/AKT/p70S6K/LARP6-associated signaling [14,43,54]. Because mTOR itself was not directly measured in that study, it provides indirect evidence for an mTOR-related anabolic branch rather than direct confirmation of mTOR-mediated quality regulation.

Taken together, AMPK-, PPAR-, and mTOR-related signaling can be viewed as recurrent regulatory nodes linking three major components of flesh quality formation: cellular energy status, lipid remodeling, and structural growth. AMPK-associated responses connect nutrient and energy sensing with substrate allocation and metabolic resilience [62,76,77], PPAR-associated pathways coordinate fatty-acid uptake, oxidation, deposition, and oxidative adaptation [69,78,79], and mTOR-related responses may participate in nutrient-sensitive protein synthesis, fiber growth and collagen-associated structural regulation [61,80]. Their relative contributions vary among species, tissues, developmental stages, and nutritional contexts, indicating that fish flesh quality formation is governed by interacting regulatory networks rather than a single linear pathway.

Foodomics can complement conventional quality evaluation by connecting molecular responses with defined quality endpoints, such as fatty-acid nutritional value, oxidative stability, flavor precursors, collagen organization, texture and water retention [28,31]. Apparently similar phenotypes may arise through different molecular routes and may consequently respond differently to nutritional, environmental, or post-harvest interventions [14,18,43,54]. Nutritional enrichment can increase oxidative susceptibility, improvements in texture may coincide with shifts in flavor-related metabolites, and structural growth requires coordinated energy and nutrient allocation. Fish flesh quality is therefore more appropriately understood as an integrated biological state in which nutritional value, texture, flavor potential, oxidative stability, and post-harvest performance emerge from interacting physiological and molecular processes rather than from isolated quality traits.

Host metabolic regulation is also intertwined with microbial processes. The intestinal microbiome can influence nutrient transformation, lipid metabolism, microbial-metabolite production, and gut–liver signaling in fish [81], providing an additional route through which pre-harvest physiological state may contribute to variation in muscle composition and flesh quality phenotypes. After harvest, product-associated microbial communities operate in a distinct biological context, where microbial metabolism contributes increasingly to biochemical deterioration, texture loss, off-odor formation, and spoilage.

## 5. Microbiome-Mediated Quality Formation, Spoilage and Preservation in Fish

**Figure 2:**
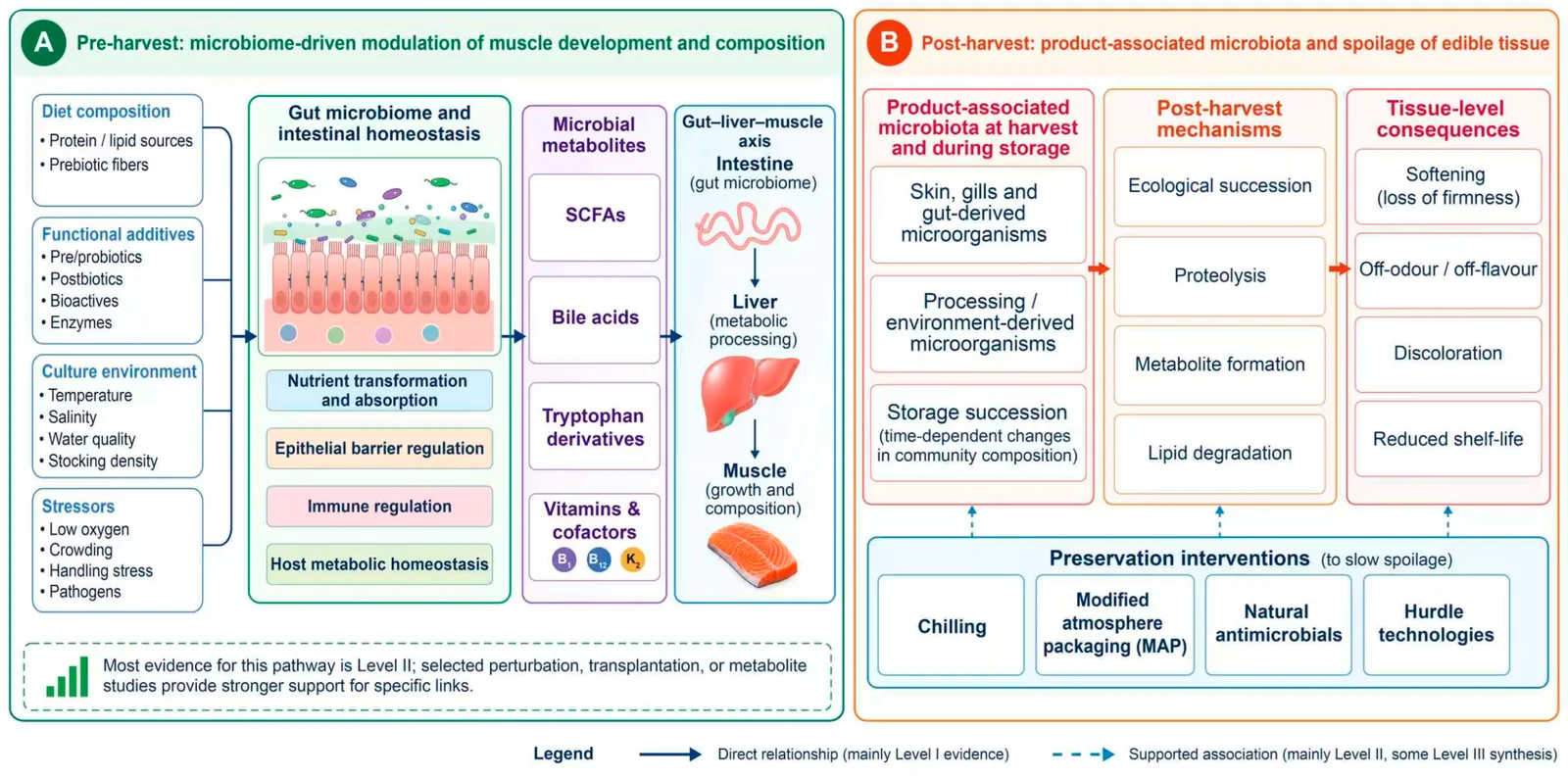
Microbiome-mediated regulation, spoilage and preservation of fish flesh quality. (**A**) Pre-harvest gut-associated modulation of muscle development and composition; (**B**) post-harvest product-associated microbial deterioration and preservation interventions. Solid arrows denote relationships demonstrated within feeding, perturbation, inoculation, storage, or preservation studies, primarily corresponding to Level I evidence. Dashed arrows denote biologically supported associations or cross-study synthesis corresponding mainly to Levels II–III.

Microbial influences on fish flesh quality operate at two biologically distinct stages. Before harvest, the intestinal microbiome participates in nutrient transformation, epithelial and immune regulation, microbial-metabolite production, and host metabolic homeostasis, providing an upstream route through which muscle development and composition may be influenced [35,36]. Environmental, dietary, and intervention studies increasingly connect intestinal microbial responses with host metabolism and edible-muscle traits [38,82,83,84]. After harvest, product-associated microorganisms act directly on edible tissue through ecological succession, proteolysis, substrate utilization, and metabolite production [43,44]. The intestinal microbiome and the microbiota associated with harvested fish therefore represent distinct microbial compartments with different substrates, functions, and relationships to flesh quality. Figure 2 summarizes these two microbial dimensions and their respective roles in pre-harvest quality formation and post-harvest deterioration.

### 5.1. Gut Microbiome and Intestinal Homeostasis as Modulators of Pre-Harvest Quality Formation

The gastrointestinal tract is a major interface through which dietary and environmental inputs are translated into host physiological responses. Fish-associated intestinal microorganisms participate in nutrient transformation, digestive function, epithelial development and immune regulation [35,36], while microbiota- or probiotic-based interventions in fish and shrimp have been associated with carbohydrate, lipid, protein and amino-acid metabolism [35,36]. These functions are relevant to flesh quality formation because muscle growth and composition depend on the digestion, absorption and systemic allocation of amino acids, fatty acids, glucose and micronutrients. Gut microbiota may therefore influence digestive and absorptive efficiency, nutrient uptake and storage, lipid metabolism and host energy homeostasis [35,36,37]. Conversely, intestinal barrier disruption may increase host exposure to pathogens or microbial products, impair nutrient absorption and promote inflammatory or metabolic disturbance, thereby weakening the physiological conditions required for muscle accretion and quality formation [85,86].

Environmental and dietary studies show that intestinal microbial ecology, host metabolism, and edible-muscle traits can shift in a coordinated manner under defined production conditions. In a rice–fish coculture system, rice flowering was associated with changes in tilapia intestinal microbiota, liver metabolites and muscle nutritional composition, including total umami amino acids and monounsaturated fatty acids [84]. Dietary proanthocyanidins improved antioxidant status and modified muscle fatty-acid and bioactive-compound profiles in common carp while restructuring intestinal microbiota and metabolic functions [82]. Galacto- and xylo-oligosaccharide supplementation in Nile tilapia altered intestinal microbiota and metabolites together with muscle amino-acid composition and texture-related properties [83]. More recently, whole-intestinal microbiota transplantation in Yellow River carp reshaped the gut microbiome and metabolic profile, improved muscle texture, reduced fat deposition and increased collagen levels, with *Cetobacterium somerae* and its metabolite acetic acid identified as candidate contributors to these muscle-quality changes [38]. Collectively, these studies place intestinal microbial ecology within a broader physiological network linking production conditions, host metabolism, and edible-muscle phenotype.

A functional interpretation of the intestinal microbiome is therefore more informative than focusing solely on the enrichment of presumed beneficial taxa. Taxonomic responses are species-, diet-, and environment-dependent, and similar taxa may perform different metabolic functions under different ecological conditions [87,88]. Microbial substrate transformation, metabolite production, and host–pathway interactions provide a more direct biological connection with muscle growth, lipid deposition, collagen turnover, redox balance, and other flesh quality traits [38,88]. Integrating microbiome profiles with texture, water-holding capacity, fatty-acid composition, flavor-related metabolites, oxidative stability, and histological traits therefore provides greater biological resolution than taxonomic profiling alone.

### 5.2. Microbial Metabolites Link Intestinal Ecology, Host Metabolism and Muscle Traits

A metabolite-centered perspective shifts microbiome research from asking which microorganisms are present to asking which biochemical functions are produced and how these functions interact with the host. This distinction is important because microbial taxa can vary substantially across fish species, diets, and environments, whereas metabolic outputs more directly reflect substrate transformation and host-interaction potential [87,88]. Fish studies indicate that intestinal microbial metabolites are associated with hepatic glucolipid metabolism, muscle collagen regulation, lipid deposition, and flesh quality traits [38,39,40,41,42,43,82,83,84,89,90,91,92,93,94,95,96,97]. In fish, short-chain fatty acids, bile acids, and microbially supplied vitamins have emerged as functional mediators of gut–host communication [41,43,91,92,93,94,95,97].

Fine-scale gastrointestinal profiling and microbiome–metabolome analyses further show that intestinal microbial ecology is closely coupled with luminal metabolic functions [89,90]. This coupling provides a biological interface between microbial substrate transformation and host pathways relevant to muscle development, nutritional composition, and post-harvest biochemical stability. Among these signals, short-chain fatty acids and bile acids currently provide particularly interpretable links between intestinal microbial metabolism and host metabolic regulation.

In fish, acetate and propionate produced or associated with intestinal microbiota have been linked with collagen synthesis, lipid metabolism, flesh texture, and other muscle-quality phenotypes [38,39,40,41,42,95]. These processes are relevant to fish flesh quality formation because muscle growth, lipid deposition, and post-mortem biochemical behavior depend on digestive efficiency, nutrient uptake, and host energy homeostasis [35,36,37]. In fish, dietary bile acids can influence FXR-associated lipid metabolism, intestinal barriers, gut microbiota, hepatic status, and muscle lipid deposition [37,91,92,93,97]. Bile-acid-related interventions have affected intestinal morphology and barrier function in largemouth bass [91], intestinal microbiota, bile-acid metabolism, and hepatic status in hybrid grouper [92], and growth, glucose metabolism, intestinal morphology, and antioxidant responses in spotted seabass under high-temperature conditions [93]. Together, these studies position SCFAs and bile-acid signaling at the interface between intestinal ecology and systemic metabolic regulation. Other microbial products further broaden the functional scope of gut–host communication. *Cetobacterium* has been shown to contribute vitamin B12-related functions and influence intestinal redox conditions and pathogen resistance in fish [94]. Although these functions are not equivalent to direct flesh quality effects, they contribute to the metabolic and physiological context within which muscle growth and composition are established.

Evidence connecting microbial metabolites with edible-muscle traits has become more direct through perturbation and mechanism-oriented studies. Whole-intestinal microbiota transplantation in Yellow River carp reshaped gut microbial and metabolic profiles and was associated with improved muscle texture, reduced fat deposition, and increased collagen levels [38]. In tilapia, the gut commensal *Cetobacterium somerae* produced acetate that promoted collagen synthesis through SMAD2/3 acetylation, and acetate supplementation increased muscle hardness and collagen deposition [39]. Another tilapia study identified a microbiota–L-glutamine–α-ketoglutarate route that facilitated collagen cross-linking and texture regulation [40]. Fermented soybean meal altered gut microbiota and propionate concentration together with changes in host lipid metabolism and flesh texture [41]. Probiotic supplementation in *Coilia nasus* likewise linked intestinal microbiota and metabolites with glucolipid metabolism, growth performance, and muscle nutritional value through a gut–liver-associated pathway [42].

The gut–liver–muscle axis provides a useful biological context for integrating these microbial and metabolic processes with foodomics. Dietary and environmental inputs can reshape intestinal microbial communities and metabolic functions [89,90]. An integrated gut–liver–muscle interpretation is supported by fish studies jointly profiling intestinal microbiota, liver metabolism, and muscle quality [43,82,83,84,95,96,98], while intervention studies identify candidate microbial-metabolite routes affecting collagen and texture [38,39,40,41,42]. Studies including edible tissues support this integrated view: rice–fish coculture linked culture environment with intestinal microbiota, liver metabolism, and muscle nutrient composition [84]; dietary proanthocyanidins connected intestinal microbial restructuring with antioxidant metabolism and muscle fatty-acid characteristics [82]; and oligosaccharide supplementation linked intestinal microbiota and metabolite changes with muscle texture and nutrient composition [83]. Together with microbiota transplantation and metabolite-focused studies [38,39,40,41,42], these findings support a metabolic continuum connecting intestinal ecology with host physiology and edible-muscle phenotype.

### 5.3. Product-Associated Microbiomes as Direct Drivers and Intervention Targets of Quality Deterioration

After harvest, the role of microorganisms changes fundamentally. Product-associated microorganisms become direct drivers of deterioration by exploiting muscle proteins, lipids, nucleotides, and nitrogenous compounds as substrates, thereby contributing to freshness loss, off-odor formation, softening, and shelf-life termination [43,44]. Total viable count remains useful as a protocol-defined culture-based indicator, but spoilage behavior depends more directly on the ecological selection of particular organisms and their functional traits under defined storage conditions. Temperature, oxygen availability, packaging atmosphere, and processing treatments shape the dominant microbial consortium, as illustrated by anchovy storage under aerobic, vacuum, and modified-atmosphere conditions [99]. Similarly, high-CO_2_ refrigerated storage delayed overall deterioration in large yellow croaker but favored Shewanella as a principal residual spoiler [43,44]. Reviews of fish storage and nonthermal preservation further show that spoilage trajectories depend strongly on product matrix, storage environment, and processing technology [30,100].

Two major functional processes explain how storage-associated microbiomes are translated into quality deterioration: structural protein degradation and spoilage-metabolite production. Controlled inoculation in grass carp showed that Pseudomonas putida promoted degradation of myofibrillar and cytoskeletal proteins, whereas *Shewanella putrefaciens* strongly affected collagen and filament-associated proteins; these changes were accompanied by myofiber disruption, softening, and altered water distribution [43,44]. Whole-genome and challenge evidence further demonstrated that *Pseudomonas psychrophila* and *S. putrefaciens* possess strong fish-protein degradation capacities, with *P. psychrophila* causing more pronounced texture deterioration and carrying serine-protease-associated genes potentially involved in extracellular protein hydrolysis [101]. Additional challenge-test evidence in fish fillets further supports the relevance of Pseudomonas spp. as spoilage organisms in real product matrices [102]. These findings show that post-harvest structural deterioration reflects the functional activity of selected microbial populations rather than simply an increase in total bacterial abundance.

In parallel, spoilage microorganisms transform endogenous muscle substrates into compounds directly associated with sensory deterioration. Amino acids, nucleotides, trimethylamine oxide, and other nitrogenous compounds can be converted into volatile bases, sulfur-containing compounds, biogenic amines, and other off-odor or off-flavor metabolites [44,99,103]. Biogenic amines are widely used as indicators of fish freshness and spoilage progression. Metagenomic and metabolomic studies further reveal organism-specific deterioration routes, including Shewanella-associated arginine decarboxylation and putrescine production in large yellow croaker [43,44], as well as temperature- and packaging-dependent genes related to biogenic amine, H_2_S, and trimethylamine formation in anchovies [99]. Spoilage can therefore be understood as a functional sequence in which storage conditions select particular microbial communities, those communities exploit available substrates, deterioration-related metabolites accumulate, and sensory or technological rejection ultimately occurs.

Microbial spoilage and oxidative deterioration often proceed together but represent partly distinct mechanisms of quality loss. Lipid and protein oxidation can alter membrane integrity, protein conformation, flavor, and nutritional value independently of microbial growth [23], whereas microbial proteolysis proceeds concurrently with oxidative deterioration during storage [23,43,44]. Plasma-activated lactic acid treatment of chilled pufferfish illustrates this partial decoupling: the treatment suppressed bacterial growth and Pseudomonas, delayed increases in TVB-N, pH, K-value, and biogenic amines, and extended shelf life, while lipid oxidation changed only minimally during the first eight days [104]. Storage and processing technologies can therefore be interpreted as both ecological and biochemical interventions that modify spoilage functions and deterioration pathways rather than merely reducing microbial counts.

The integration of metagenomics, metabolomics, texture analysis, oxidation indices, and sensory evaluation provides a more complete description of preservation effects and spoilage progression [30,100]. Such integration can distinguish whether a treatment acts primarily through microbial suppression, modification of spoilage metabolism, protection of structural proteins, oxidative control, or combinations of these processes. Viewed together, the pre-harvest intestinal microbiome and the post-harvest product-associated microbiome represent two distinct but complementary microbial dimensions of fish flesh quality: the former participates in host metabolic and structural regulation, whereas the latter directly contributes to biochemical deterioration, structural damage, and spoilage after harvest.

## 6. From Quality Prediction to Mechanism-Informed Intelligent Management

The rapid development of spectroscopy, artificial sensing, computer vision, and machine learning has transformed fish-quality assessment from conventional endpoint measurement toward rapid, nondestructive, and increasingly automated evaluation [24,59,60]. Across these technologies, the dominant modeling paradigm remains broadly similar: instrumental signals are mapped onto predefined outcomes such as freshness grade, storage stage, TVB-N, K-value, microbial load, or other quality indices [59,105]. These models can substantially improve analytical efficiency, but accurate prediction of an endpoint does not necessarily reveal the biological processes that produced it. Foodomics provides molecular-level information on metabolic, oxidative, enzymatic, and microbial changes associated with fish-quality deterioration [30], whereas intelligent sensing systems primarily capture optical, volatile, electrical, spectral, or image-based manifestations of these processes [59,60]. The emerging challenge is therefore to combine predictive performance with biological interpretation, transferability, and relevance to practical quality-management decisions.

This challenge becomes particularly important when pre-harvest quality formation and post-harvest deterioration are considered as parts of the same biological continuum. Before harvest, flesh quality is shaped by nutrition, physiological state, rearing environment, muscle growth, and host-associated biological regulation [56,98,106]. After harvest, quality change increasingly reflects ATP depletion, mitochondrial dysfunction, myofibrillar degradation, microbial metabolism, oxidation, and structural destabilization [18,53,107]. These stages are biologically connected because muscle entering storage retains energetic, structural, lipid, redox, and microbial characteristics established at harvest, but they represent different analytical and management problems. Models intended to optimize quality formation, predict shelf life, or guide preservation therefore require different targets and biological information. Mechanism-informed quality intelligence must distinguish how quality is formed, what biological state exists at harvest, how that state subsequently evolves, and which deterioration process becomes limiting under a given post-harvest context.

### 6.1. From Quality-State Recognition to Prospective Prediction

Current intelligent assessment of fish quality is most mature in recognizing the present product state. HSI, Raman spectroscopy, electronic sensing, and image-based deep learning detect different manifestations of quality change and therefore interrogate different layers of the flesh quality phenotype [105,108,109,110,111]. HSI combines spectral and spatial information, enabling both storage-state discrimination and visualization of within-fillet heterogeneity [105]. Raman spectroscopy provides chemically sensitive molecular fingerprints for freshness assessment [108], whereas image-based deep learning captures deterioration-associated visual changes in anatomical regions such as the eyes and gills [109]. Multimodal spectroscopy and artificial sensing further expand this coverage by integrating optical, volatile, taste, and color responses [107,108,109,110,111,112,113,114,115,116,117,118,119,120].

The transition from single-modal sensing to multimodal sensing has increased the breadth of information available to predictive models. Fusion of visible–near-infrared (VIS–NIR), short-wave infrared (SWIR), and fluorescence information improved freshness discrimination compared with individual sensing modes [110], while combined electronic-nose (E-nose), electronic-tongue (E-tongue), and color information enabled simultaneous estimation of K-value, lipid oxidation, protein oxidation, and calcium-dependent adenosine triphosphatase (Ca^2+^-ATPase) activity [111]. Multimodal sensing therefore provides a broader representation of product state, although most prediction targets still correspond to freshness classes or physicochemical changes that have already emerged. These technologies can consequently be viewed along a continuum from visually observable phenotypes to increasingly chemistry-sensitive proxy measurements rather than as interchangeable solutions to a single classification problem.

A more important transition occurs when prediction moves from the current state toward future quality trajectories and functional outcomes. Multi-species shelf-life modeling has extended prediction from present freshness to the expected duration of acceptable quality under different temperature conditions. Cross-species fluorescence modeling further indicates that part of the learned quality information can be retained across species after fine-tuning [113]. Prospective prediction has also expanded beyond shelf-life estimation: frozen-state hyperspectral imaging can predict thaw-induced drip loss before visible exudation occurs [114], while hyperspectral imaging combined with temporal deep learning can continuously track gel strength, water-holding capacity, and whiteness during surimi heating [115]. These developments mark a shift from static classification toward models that incorporate time, transferability, and evolving product function.

Model generalization remains central to this transition. Performance obtained under controlled experimental conditions may change across production batches, biological backgrounds, instruments, processing environments, or commercial settings, making validation design and calibration transfer essential to model deployment [116,117,118]. Prospective prediction also differs from mechanistic interpretation: a model may predict remaining shelf life, drip loss, or processing behavior accurately without identifying the biological process that determines the outcome.

The biological meaning of model inputs therefore becomes increasingly important as prediction moves closer to management. Storage day, TVB-N, TVC, and K-value are closely associated with the quality outcomes they represent, whereas spectral bands, image features, and artificial-sense outputs generally act as indirect proxies for underlying biochemical or structural changes. Molecular and microbial measurements directly characterize metabolic and microbial processes associated with deterioration [30,107,119]. Volatolomics combined with machine learning, for example, can reduce high-dimensional chemical profiles to candidate freshness-associated compounds and related metabolic pathways [119], while multi-omics analyses can distinguish spoilage trajectories associated with different microbial and metabolic states [107]. Integrating such biologically informative features with sensing data provides a route from quality-state recognition toward prediction that is both statistically robust and biologically interpretable. Representative analytical and predictive models illustrating this progression from current-state recognition to prospective quality prediction are summarized in Table 2.

### 6.2. Linking Pre-Harvest Quality Formation to Post-Harvest Fate Through the Harvest Biological State

Pre-harvest flesh quality is a multidimensional phenotype shaped by interacting nutritional, physiological, environmental, structural, and microbiome-associated processes [41,56,95,106]. Production interventions can simultaneously alter texture, water-holding capacity, lipid deposition, flavor, and nutritional composition, but these traits do not necessarily respond in parallel [6,10,56,121]. Microbial and metabolic changes have been associated with muscle-quality phenotypes across aquaculture systems [95,96,98], while propionate and other short-chain fatty acids have been implicated in lipid metabolism, collagen regulation, and flesh quality responses in fish [41,43,95]. Pre-harvest quality is therefore more appropriately represented as a multidimensional biological profile than as a single high-to-low-quality score.

This interpretation changes the role of artificial intelligence in production-stage quality management. Rather than simply classifying fish into predefined quality categories, predictive systems can relate controllable production conditions to multiple biologically meaningful traits. Live-fish hyperspectral imaging already permits nondestructive estimation of intrinsic texture characteristics [120], while physics-informed aquaculture modeling provides a computational precedent for incorporating biological constraints into data-driven prediction [122]. Together, these developments provide a basis for moving from single-trait classification toward biologically informed, multi-trait decision support.

The critical interface between quality formation and post-harvest fate is the biological condition of muscle and product at harvest. Farming stress, fasting, transport, and slaughter can modify energy metabolism, muscle structure, and water retention before death [10,123]. Pre-harvest conditions can also influence redox state and subsequent oxidative susceptibility [124], while the microbial condition of the harvested product can affect later spoilage trajectories [107,125]. To organize these interacting dimensions, we propose a context-dependent harvest biological-state vector (HBSV): HBSV = [E, S, L, R, M].

Above, E represents energetic state, S structural state, L lipid and nutritional state, R redox state, and M the product-associated microbial starting state. The HBSV describes the multidimensional biological condition from which post-harvest change begins. Its operational measurements may therefore differ among fish species, product forms, production systems, and intended management applications. The pre-harvest intestinal microbiome is treated as an upstream regulatory layer, whereas the microbial HBSV domain refers specifically to the microbiota associated with the harvested product unless transfer to edible tissue is demonstrated.

The biological rationale underlying these HBSV dimensions is supported by intervention, comparative multi-omics, post-mortem, and longitudinal storage studies. Representative evidence linking production conditions, biological processes, and quality outcomes is summarized in Table 3.

After harvest, endogenous enzymatic activity, oxidation, structural deterioration, and microbial growth proceed concurrently, while their relative importance varies with product characteristics and exposure conditions [53,100,125]. The biological state present at harvest may therefore influence not only initial quality but also the rate and pathway of subsequent deterioration. Energetic status can affect ATP depletion, rigor development, and pH change; structural condition influences water retention and susceptibility to texture loss; lipid composition determines both nutritional value and oxidation substrate availability; redox state influences oxidative susceptibility; and the product-associated microbial starting state contributes to the ecological conditions from which spoilage communities develop. These domains are not intended to function as independent quality scores. Rather, together they provide a structured representation of the biological starting condition against which later deterioration can be interpreted. Current-state sensors and conventional indicators remain useful for monitoring post-harvest change, whereas prospective management additionally requires variables that predict deterioration rate, time to unacceptable quality, and the dominant mode of quality failure. Candidate measurements, practical surrogates, and validation requirements for operationalizing each HBSV domain are summarized in Table 4.

### 6.3. Reliability, Generalization and Interpretability of Intelligent Quality Models

The transition from benchmark prediction to deployable quality intelligence begins with validation design. Multiple spectra, pixels, image patches, or technical replicates derived from the same fish should not be distributed across both training and test sets. The biological individual therefore represents the minimum unit for train–test separation, while grouped splitting at the level of batch, cage, farm, or processing lot may be required when samples share higher-level dependencies. Preprocessing, dimensionality reduction, feature selection, and hyperparameter tuning should be conducted exclusively within training data, preferably using grouped or nested cross-validation, to minimize data leakage [116,117].

Internal accuracy or R^2^ should be complemented by metrics appropriate to the prediction target, including sensitivity, specificity, balanced accuracy, mean absolute error, root mean square error, calibration, confidence intervals, and uncertainty estimates. More importantly, external validation should progressively extend across independent fish, batches, farms, seasons, processing plants, instruments, and analytical platforms. Calibration transfer or model adaptation may be required when measurement conditions change [118,126]. Generalization is therefore demonstrated by performance on biologically and operationally independent samples rather than by random splitting of correlated measurements.

Interpretability provides an additional layer of information but does not by itself establish a biological mechanism. XAI methods can identify influential variables, spectral regions, or image features [51,127,128]. Features with high Shapley additive explanations (SHAP), local interpretable model-agnostic explanations (LIMEs), or saliency importance remain predictive signals until their biological relevance is supported by independent biochemical, physiological, microbial, structural, or intervention evidence. Mechanism-informed interpretation consequently progresses from statistical attribution to biological concordance and, where possible, experimental validation.

Prediction reliability also depends on recognizing when a model is uncertain or operating outside its calibration domain. Knowledge-guided modeling can reduce variance associated with measurement orientation [129], while conformal prediction provides a route to sample-specific prediction intervals with controlled statistical properties [130]. Predictions from uncertain or out-of-domain samples should therefore carry different decision weights from estimates obtained within well-represented calibration space. External validity, calibration stability, interpretable feature use, quantified uncertainty, and biological plausibility jointly determine whether an intelligent model can support fish-quality management [52,116,117,118,126,129,130].

### 6.4. A Dual-Stage Framework Linking Quality Formation to Post-Harvest Fate

**Figure 3:**
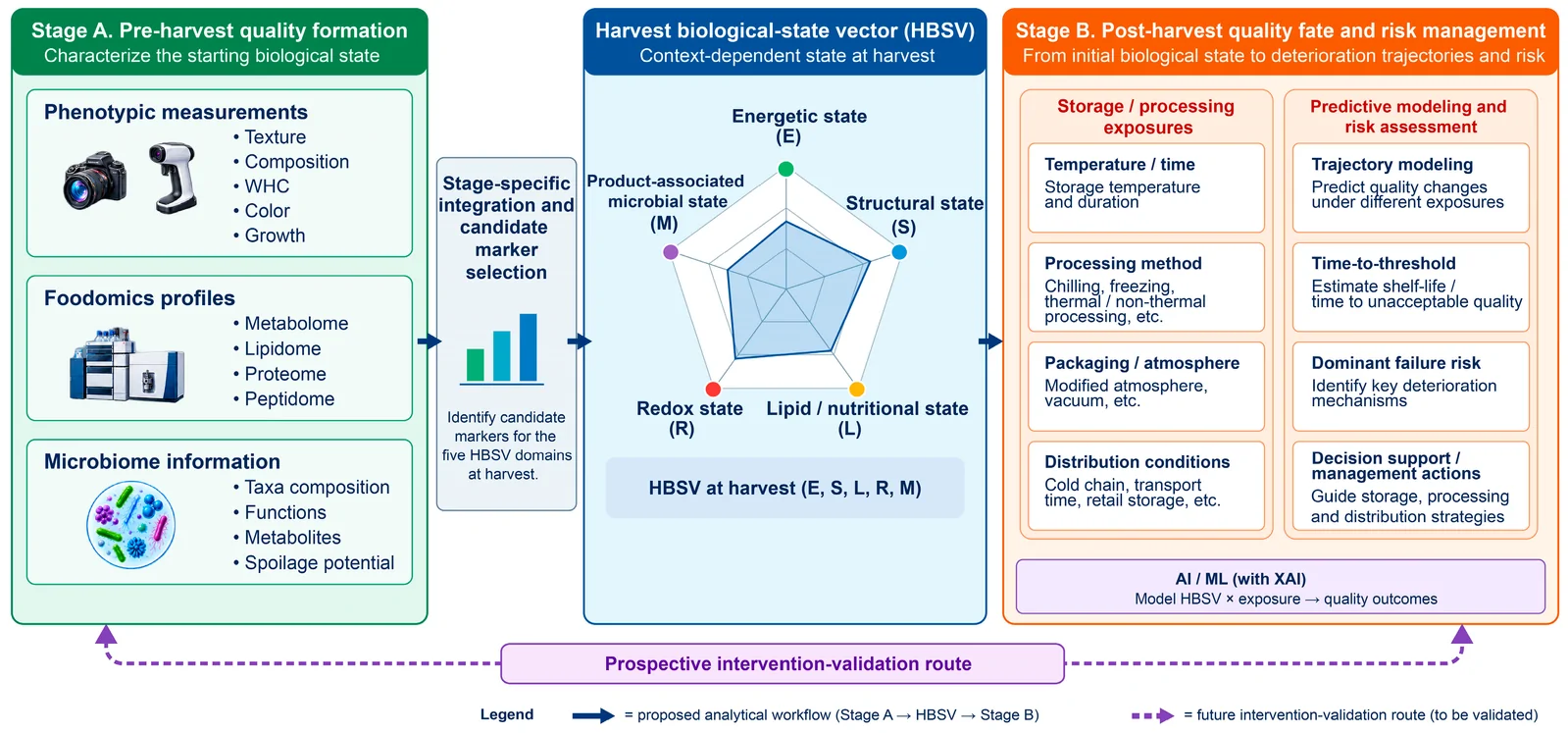
Proposed dual-stage framework for mechanism-informed fish flesh quality management. The HBSV links Stage A, which concerns controllable formation of a multidimensional quality profile, with Stage B, which concerns post-harvest deterioration trajectories and risk management. The five HBSV domains are energetic, structural, lipid/nutritional, redox, and product-associated microbial state. Solid workflow arrows indicate the analytical sequence, whereas dashed feedback arrows represent intervention–validation routes proposed for prospective testing.

Building on the evidence synthesized above, we propose a dual-stage framework that links pre-harvest quality formation with post-harvest quality fate through the HBSV (Figure 3). Phenotypic measurements, foodomics profiles, and microbiome information represent complementary data layers with distinct biological meanings. Their stage-specific integration can be used to identify candidate indicators for the energetic, structural, lipid/nutritional, redox, and product-associated microbial domains at harvest. This biological starting state can then be considered together with storage and processing exposures to model deterioration trajectories, time-to-threshold, and dominant quality risks.

In the pre-harvest stage, AI can be used to identify how controllable production factors and associated biological processes shape combinations of texture, water-holding capacity, nutritional composition, flavor, and oxidative stability. Phenotypic variables define measurable quality outcomes; foodomics data provide molecular and metabolic information; and microbiome data characterize microbial functions and metabolites associated with host metabolism and muscle development [6,41,56,95,106,121]. Rather than combining all variables indiscriminately in a single high-dimensional model, a progressive marker-selection strategy can integrate these layers. Cross-layer analysis first identifies relationships among phenotypes, molecular pathways, and microbial functions; feature-selection and explainable models then prioritize stable predictors; and concordance across data layers narrows the candidate set toward biologically coherent markers. Independent production experiments can subsequently determine whether variation in controllable production factors is accompanied by the expected changes in flesh quality traits and harvest biological state.

Post-harvest modeling addresses a different question: how the inherited HBSV interacts with storage and processing conditions to determine subsequent deterioration. Temperature, oxygen availability, packaging atmosphere, freezing–thawing history, and handling conditions define the external exposure context, whereas phenotype, foodomics, and microbiome measurements characterize the evolving product state. Phenotypic indicators describe texture loss, drip loss, color change, and freshness; foodomics resolves oxidative, proteolytic, and metabolic alterations; and microbiome data characterize spoilage-community succession and functional potential [30,53,107,119,124,125]. AI can then model time-dependent trajectories and distinguish whether microbial spoilage, oxidative instability, or structural deterioration is likely to become the dominant limiting process. Marker selection at this stage therefore prioritizes variables associated with deterioration rate, time-to-threshold, and failure mode rather than variables that merely separate fresh from spoiled products.

The continuity of the framework lies in the dual role of the HBSV. Before harvest, it represents the biological state shaped by production conditions; after harvest, it becomes the initial condition from which deterioration trajectories emerge. AI and XAI provide analytical tools for reducing high-dimensional phenotypic, foodomics, and microbiome information into stable and interpretable marker sets, while independent validation determines whether these markers retain predictive value across batches, seasons, farms, instruments, and production systems [51,52,116,117,118,126,130]. A mechanism-informed management sequence can therefore progress from biological characterization to prospective prediction and intervention testing: reproducible HBSV markers are identified, their value for predicting post-harvest trajectories is evaluated, and targeted interventions can then be tested against the deterioration process predicted to be limiting. In this way, quality formation, harvest biological state, post-harvest trajectory, and management response are connected within a common analytical framework.

## 7. Conclusions and Outlook

Current evidence supports fish flesh quality as a multidimensional biological phenotype that begins to form before harvest through coordinated metabolic, lipid, redox, structural, and microbiome-associated processes and continues to evolve after harvest through biochemical, oxidative, structural, and microbial deterioration. Conventional quality measurements remain indispensable because they provide robust phenotypic reference points and, in many cases, biologically informative readouts. Foodomics extends this information by resolving molecular pathways associated with quality formation and deterioration, whereas microbiome analysis adds a complementary layer by linking microbial functions and metabolites with host-associated regulation before harvest and direct spoilage processes after harvest. Together, these approaches support a shift from isolated quality indicators toward an integrated understanding of fish flesh quality as a dynamic biological state.

On the basis of this evidence, we propose that intelligent sensing and machine learning can extend the framework by enabling rapid quality recognition, prospective prediction, and integration of high-dimensional information. Their greatest value lies not simply in improving classification accuracy but in connecting measurable signals with biologically meaningful processes and management-relevant outcomes. The proposed dual-stage framework links pre-harvest quality formation with post-harvest quality fate through the HBSV, which organizes energetic, structural, lipid/nutritional, redox, and product-associated microbial information into a common representation of the biological condition at harvest. This proposal provides a conceptual bridge between production-stage regulation and post-harvest trajectory prediction while allowing different species, products, and management objectives to be represented through context-specific marker combinations.

This proposal requires prospective experimental validation before it can support operational management. Priority should be given to identifying minimal and biologically coherent HBSV marker panels, determining whether these markers improve prediction of post-harvest trajectories beyond conventional baselines, and testing whether interventions targeted to the predicted dominant deterioration process modify subsequent quality outcomes. Robust validation across independent fish, batches, seasons, production systems, processing environments, instruments, and analytical platforms will be essential for establishing transferability and practical utility. The longer-term goal is to develop mechanism-informed systems that connect biological state, future quality trajectory, and targeted intervention within a unified framework for intelligent fish flesh quality management.

## Figures and Tables

**Table 1:** Practical applications and biological information provided by conventional fish flesh quality assessment approaches.

Approach	Representative Indicators or Technologies	Primary Quality Dimension	Practical Strengths and Applications	Biological Information
Sensory and freshness assessment	Quality Index Method (QIM), sensory scoring	Freshness, odor, appearance, flavor, overall acceptability	Directly relevant to consumer perception; widely used for shelf-life evaluation and species-specific freshness grading	Integrates sensory consequences of autolysis, nucleotide degradation, oxidation, microbial growth, and storage-related deterioration [15,16,17]
Texture analysis	Hardness, springiness, cohesiveness, chewiness, shear force	Firmness, tenderness, eating quality, processing performance	Rapid, standardized, and widely applicable for comparing species, dietary treatments, production systems, and processing responses	Reflects muscle-fiber architecture, collagen organization, extracellular-matrix properties, myofibrillar integrity, proteolysis, and post-mortem structural changes [6,7,43,54]
Water-holding capacity	Drip loss, cooking loss, thawing loss	Juiciness, yield, structural stability	Simple, reproducible, and directly relevant to processing yield, product appearance, and consumer quality	Reflects interactions among pH, protein integrity, tissue structure, extracellular matrix, and water distribution within muscle [8,43,54]
pH and biochemical freshness indicators	pH, ATP degradation, K-value, total volatile basic nitrogen (TVB-N)	Post-mortem biochemical state and freshness	Widely used for monitoring post-mortem progression, storage deterioration, and shelf-life changes	Provides information on glycolysis, acidification, ATP depletion, nucleotide turnover, rigor development, and biochemical deterioration [15,18,19]
Color and appearance assessment	Lightness (L*), redness–greenness (a*), and yellowness–blueness (b*); image analysis	Visual quality and marketability	Rapid and potentially nondestructive; suitable for grading, quality monitoring, and industrial screening	Reflects pigment status, oxidation reactions, browning processes, tissue characteristics, and storage-associated changes [21,24]
Nutritional composition analysis	Protein, total lipid, amino acids, fatty acids	Nutritional value and compositional quality	Provides direct assessment of nutrient deposition and nutritional characteristics; useful for evaluating dietary and environmental effects	Reflects nutrient allocation, protein and lipid deposition, amino-acid composition, and fatty-acid profiles associated with muscle composition [12,20,22]
Lipid and redox assessment	Eicosapentaenoic acid (EPA), docosahexaenoic acid (DHA), PUFA/SFA ratio, lipid classes, thiobarbituric acid-reactive substances (TBARSs), protein carbonyls	Nutritional lipid quality and oxidative stability	Useful for evaluating nutritional value, flavor potential, and susceptibility to oxidative deterioration during storage	Provides information on fatty-acid remodeling, lipid/protein oxidation, antioxidant status, and redox balance [12,22,23]
Nondestructive sensing technologies	Hyperspectral imaging, Raman spectroscopy, near-infrared spectroscopy, computer vision, electronic nose, electrical impedance spectroscopy	Rapid phenotyping, freshness, composition, texture, and processing-related traits	High throughput, minimal sample destruction, and potential for online grading, screening, and process monitoring	Captures chemical, structural, spectral, spatial, volatile, or electrical proxies associated with underlying quality-related changes [15,24,25,26,58,59,60]

**Table 2:** Representative predictive and analytical models for fish flesh quality assessment.

Species or System	Analytical Input	Prediction Target	Biological Unit or Context	Validation Design	Reported Performance	Reference
Atlantic salmon	HSI, 400–1000 nm	Storage state or freshness	Fillet; repeated spatial measurements	Internal classification	Mean accuracy 77.0%	Hardy et al. [105]
Sea bass	Portable Raman spectra	Freshness grade	Fish-product sample	Convolutional neural network (CNN)-based validation	Accuracy 90.6%	Wang et al. [108]
Seabream	Eye and gill images	Fresh, intermediate, or spoiled	Fish images	Deep-learning classification with gradient-weighted class activation mapping (Grad-CAM) and local interpretable model-agnostic explanations (LIME)	High classification performance	Genç et al. [109]
Salmon and sablefish	Visible–near-infrared (VIS–NIR), short-wave infrared (SWIR), and fluorescence	Freshness state	Product samples	Multimodal comparison	Fusion outperformed single modes	Kashani Zadeh et al. [110]
Horse mackerel	Electronic nose (E-nose), electronic tongue (E-tongue), and color	K-value, thiobarbituric acid (TBA), carbonyls, Ca^2+^-ATPase	Whole-fish samples	Regression test set	Test R^2^ 0.936–0.994	Li et al. [111]
Five marine fish species	Species, temperature, TVC, K-value, TVB-N, sensory and volatiles	Remaining shelf life	Multi-species dataset	Shelf-life model	Prediction error < 0.5 d	Cui et al. [112]
Bighead carp and rainbow trout	Excitation–emission matrix (EEM) fluorescence	TVB-N, K-value, TVC, freshness class	Cross-species samples	Transfer and fine-tuning	Accuracy > 88.4%	Fan et al. [113]
Crisp tilapia	Frozen-state visible and near-infrared (VNIR) or SWIR HSI	Post-thaw drip loss	Fish fillet	Independent prediction set	R_p_^2^ = 0.8520; root mean square error of prediction (RMSEP) = 1.8713	Tang et al. [114]
Live spotted seabass	Live-fish HSI	Texture-profile traits	Biological individual	Independent prediction set	Best R_p_^2^ > 0.90	Che et al. [120]
Surimi	HSI and convolutional neural network–long short-term memory (CNN–LSTM)	Gel strength, WHC, whiteness	Dynamic thermal process	Temporal prediction	R_p_^2^ > 0.93	Xia et al. [115]

**Table 3:** Representative biological and intervention evidence supporting the mechanism-informed framework.

Species or System	Intervention or Exposure	Biological Layer	Quality-Related Outcome	Evidence Type	Interpretation	Reference
Gibel carp	Dietary lipid and exercise	Lipid metabolism and muscle structure	Lipid, texture, WHC, IMP, EPA, and flavor traits	Level I: controlled intervention	Demonstrates multidimensional and nonparallel responses	Cai et al. [56]
Nile tilapia	Fermented soybean meal	Gut microbiome and propionate	Muscle lipid and texture	Level I–II: intervention plus association	Supports a candidate microbiome–metabolite–muscle route	Wang et al. [41]
Common carp	Rice–fish coculture	Microbiome, SCFAs, muscle metabolism	Metabolic and quality traits	Level II: comparative multi-omics	Links production environment with coordinated microbial and muscle responses	He et al. [95]
Acrossocheilus fasciatus	Four aquaculture systems	Nutrition, microbiome, metabolome	Nutritional, flavor, and quality profiles	Level II: comparative multi-omics	Shows system-dependent shifts across quality dimensions	Fan et al. [98]
Fish muscle	Post-mortem progression	ATP, mitochondria, pH, myofibrils	Rigor, texture, freshness	Level I: mechanism-oriented synthesis	Defines energetic and structural components of harvest state	Hematyar et al. [18]
Farmed fish	Feed, environment, pre-slaughter stress	Redox physiology	Antioxidant state and later oxidation	Level I–II	Supports redox state as an HBSV domain	Nathanailides and Kontominas [124]
Refrigerated salmon	Storage	Volatolomics and random forest	Freshness-associated volatile compounds	Level II: marker discovery and validation	Identifies interpretable candidate freshness markers	Hu et al. [119]
Cold-smoked salmon from three plants	Plant and storage context	Microbiome, volatiles, metabolites	Spoilage trajectories	Level II: longitudinal multi-omics	Shows divergent trajectories across starting contexts	Macé et al. [107]
Fish storage and preservation evidence	Storage and preservation conditions	Microbial, oxidative, and structural deterioration	TVC, TVB-N, oxidation, protein degradation	Synthesis and intervention evidence	Shows that the dominant deterioration process depends on product and context	Xie et al. [100]; Zhuang et al. [53]; Nie et al. [125]

**Table 4:** Operationalization and prospective validation of the proposed harvest biological-state vector.

HBSV Domain	Biological Meaning	Candidate Measurements	Current Evidence	Practical Surrogate	Required Validation
Energetic state (E)	Readiness for ATP depletion, rigor, and pH development	ATP, ADP, AMP, IMP, glycogen, lactate, pH	Relatively established	Targeted nucleotide panel plus pH	Prospective association with post-mortem trajectories across batches and temperatures
Structural state (S)	Architecture and water-retention capacity	Fiber diameter and density, collagen, sarcomere integrity, WHC, texture	Established and intervention-supported	Histology plus WHC and texture	Independent prediction of structural failure and processing performance
Lipid and nutritional state (L)	Nutritional composition and oxidation substrate	Fatty-acid profile, lipid classes, EPA/DHA, protein, amino acids	Established for composition; partial for trajectory	Targeted lipid and composition panel	Cross-species and cross-diet validation against nutritional and storage outcomes
Redox state (R)	Resistance or susceptibility to oxidative damage	Antioxidant capacity, redox enzymes, lipid and protein oxidation markers	Moderately supported	Selected antioxidant and oxidation markers	Pre-harvest-to-storage time courses and intervention tests
Microbial starting state (M)	Product-associated spoilage potential at harvest	Specific spoilage organisms, community composition, functional genes, contamination context	Emerging and context-dependent	Targeted microbial panel plus handling metadata	Longitudinal validation across farms, plants, seasons, and packaging conditions

## Data Availability

No new data were created or analyzed in this study. Data sharing is not applicable to this article.

## Abbreviations

The following abbreviations are used in this manuscript:

Abbreviation	Full Term
ADP	Adenosine diphosphate
AI	Artificial intelligence
AKT	Protein kinase B
AMP	Adenosine monophosphate
AMPK	AMP-activated protein kinase
ATP	Adenosine triphosphate
Ca^2+^-ATPase	Ca^2+^-dependent adenosine triphosphatase
CNN	Convolutional neural network
CNN–LSTM	Convolutional neural network–long short-term memory
DHA	Docosahexaenoic acid
DL	Deep learning
EEM	Excitation–emission matrix
E-nose	Electronic nose
E-tongue	Electronic tongue
EPA	Eicosapentaenoic acid
FOXO3a	Forkhead box O3a
FXR	Farnesoid X receptor
Grad-CAM	Gradient-weighted class activation mapping
HBSV	Harvest biological-state vector
HSI	Hyperspectral imaging
IGF	Insulin-like growth factor
IGF-1	Insulin-like growth factor 1
IMP	Inosine monophosphate
LARP6	La-related protein 6
LIME	Local interpretable model-agnostic explanations
LSTM	Long short-term memory
MMP-2	Matrix metalloproteinase 2
mTOR	Mechanistic target of rapamycin
p70S6K	70-kDa ribosomal protein S6 kinase
PI3K	Phosphoinositide 3-kinase
PPAR	Peroxisome proliferator-activated receptor
PUFA	Polyunsaturated fatty acid(s)
QIM	Quality Index Method
RF	Random forest
RMSEP	Root mean square error of prediction
S6K1	Ribosomal protein S6 kinase 1
SCFAs	Short-chain fatty acids
SFA	Saturated fatty acid(s)
SMAD2/3	SMAD family members 2 and 3
SREBP-1c	Sterol regulatory element-binding protein 1c
SWIR	Short-wave infrared
TBA	Thiobarbituric acid
TBARS	Thiobarbituric acid-reactive substances
TGF-β1	Transforming growth factor beta 1
TGR5	Takeda G protein-coupled receptor 5
TIMP-2	Tissue inhibitor of metalloproteinases 2
TOR	Target of rapamycin
TPA	Texture profile analysis
TVB-N	Total volatile basic nitrogen
TVC	Total viable count
VIS–NIR	Visible–near-infrared
VNIR	Visible and near-infrared
VOCs	Volatile organic compounds
WHC	Water-holding capacity
XAI	Explainable artificial intelligence
